# PtoMYB156 is involved in negative regulation of phenylpropanoid metabolism and secondary cell wall biosynthesis during wood formation in poplar

**DOI:** 10.1038/srep41209

**Published:** 2017-01-24

**Authors:** Li Yang, Xin Zhao, Lingyu Ran, Chaofeng Li, Di Fan, Keming Luo

**Affiliations:** 1 Key Laboratory of Eco-environments of Three Gorges Reservoir Region, Ministry of Education, Institute of Resources Botany, School of Life Sciences, Southwest University, Chongqing 400715, China

## Abstract

Some R2R3 MYB transcription factors have been shown to be major regulators of phenylpropanoid biosynthetic pathway and impact secondary wall formation in plants. In this study, we describe the functional characterization of *PtoMYB156*, encoding a R2R3-MYB transcription factor, from *Populus tomentosa.* Expression pattern analysis showed that *PtoMYB156* is widely expressed in all tissues examined, but predominantly in leaves and developing wood cells. PtoMYB156 localized to the nucleus and acted as a transcriptional repressor. Overexpression of *PtoMYB156* in poplar repressed phenylpropanoid biosynthetic genes, leading to a reduction in the amounts of total phenolic and flavonoid compounds. Transgenic plants overexpressing *PtoMYB156* also displayed a dramatic decrease in secondary wall thicknesses of xylem fibers and the content of cellulose, lignin and xylose compared with wild-type plants. Transcript accumulation of secondary wall biosynthetic genes was down-regulated by *PtoMYB156* overexpression. Transcriptional activation assays revealed that PtoMYB156 was able to repress the promoter activities of poplar *CESA17, C4H2* and *GT43B.* By contrast, knockout of *PtoMYB156* by CRISPR/Cas9 in poplar resulted in ectopic deposition of lignin, xylan and cellulose during secondary cell wall formation. Taken together, these results show that PtoMYB156 may repress phenylpropanoid biosynthesis and negatively regulate secondary cell wall formation in poplar.

In plants, phenylpropanoid compounds are a wide range of secondary metabolites including monolignols, flavonoids, stilbenes and various phenolic acids. These natural products are involved in mechanical strength, plant defense and ultraiolet (UV) light protectants^1^. The first three reactions in the phenylpropanoid metabolism pathway are catalyzed by phenylalanine ammonia-lyase (PAL; EC 4.3.1.5), cinnamate 4-hydroxylase (C4H; EC 1.14.13.11), and 4-coumarate: CoA ligase (4CL; EC 6.2.1.12), respectively, leading to the synthesis of *p*-coumaroyl CoA which is a common precursor for the production of many important compounds including monolignols and flavonoids^2^.

Lignin is a complex organic polymer of monolignols and constitutes one of the major components of the secondary walls of xylem cells and fibres. Secondary cell walls are the primary constituent of fibers and tracheary elements in wood, which is one of the most abundant feedstock resources in the world, and ensure water and nutrient transport and provide plants with rigidity and strength to support their body weight. Secondary wall formation is an ordered developmental process that requires the fine temporal and spatial regulation of the genes involved in the biosynthesis and targeted secretion of secondary wall components, and oriented deposition and assembly of secondary walls^3^,^4^,^5^. In the past decade, comprehensive molecular and genetic studies have revealed a complex regulatory network for secondary wall biosynthesis^5^,^6^,^7^,^8^.

A hierarchical network of transcription factors has been proposed to control secondary wall formation in plants^5^,^9^. Several NAC (for NAM, ATAF1/2, and CUC2) transcription factors including NST1/2, NST3/SND1, VND6/7 act as master switches that activate secondary wall biosynthesis in Arabidopsis (*Arabidopsis thaliana*)^5^,^10^,^11^. In woody plants such as poplar and *Eucalyptus*, a group of wood-associated NAC transcription factors (PtrWNDs and EgWND1) have been identified as functional orthologs of the Arabidopsis secondary wall biosynthesis-related NACs including SND1, NST1/2, and VND6/7^12^,^13^. Besides NAC proteins, several MYB transcription factors were also shown to be key regulators of secondary wall formation. In poplar, at least four MYB transcription factors (PtrMYB2, PtrMYB3, PtrMYB20 and PtrMYB21) have been demonstrated to be direct targets of PtrWNDs and functional orthologs of the Arabidopsis MYB46 and MYB83 which act as second-level master switches controlling secondary wall biosynthesis^4^,^14^,^15^. These PtrMYBs are able to activate the promoter activities of poplar wood biosynthetic genes^15^. When overexpressed in Arabidopsis, PtrMYB3/20 were also capable of activating the biosynthetic pathways of secondary cell wall, resulting in ectopic deposition of cellulose, xylan and lignin^14^. Other wood-associated transcription factors as master switches activated secondary wall biosynthesis during wood formation include the *Eucalyptus* EgMYB2^16^ and the pine (*Pinus taeda*) PtMYB4/8^17^,^18^. These transcription factors have been shown to be functional orthologs of *Arabidopsis* MYB46/83, and overexpression of PtMYB4 and EgMYB2 resulted in ectopic lignification in tobacco and *Arabidopsis*, respectively^13^,^17^. There are also many R2R3 MYB transcription factors that directly bind to AC *cis*-elements (AC-I, ACCTACC; AC-II, ACCAACC, and AC-III, ACCTAAC) in lignin biosynthetic gene promoters to positively and negatively regulate lignin synthesis^8^,^19^. Among of them, AtMYB58 and AtMYB63^20^, AtMYB85^6^ and AtMYB83^21^, and PtMYB1^18^ have been identified as transcriptional activators, and AtMYB4^22^, AtMYB32^23^, ZmMYB31^24^, ZmMYB42^24^, PvMYB4^26^, EgMYB1^27^, ZmMYB11^19^, VvMYBC2-L1/L3^28^ as repressors.

In the *P. trichocarpa* genome, at least 192 R2R3 MYB transcription factors have been annotated^29^. To date, increasing evidence shows the involvement of a few transcription factors in the regulation of lignin biosynthesis in poplar. PtrMYB28^30^, PtrMYB152^31^,^32^ and PtoMYB92^33^ have been reported as activators of lignin biosynthesis. However, only a few MYB transcription factors have been demonstrated to be a repressor of lignin biosynthesis in poplar. More recently, overexpression of *PdMYB221* from *P. deltoids* led to a reduction in secondary cell wall thicknesses of fibers and vessels in transgenic *Arabidopsis*^34^, indicating that PdMYB221 may be a repressor of secondary wall formation in poplar. In the present study, an R2R3 MYB gene, *PtoMYB156*, was isolated from Chinese white poplar *(P. tomentosa* Carr.) based on homology with *Arabidopsis* AtMYB4 and *Eucalyptus* EgMYB1 of known function as repressors in lignin biosynthesis. When overexpressed in poplar, PtoMYB156 is also able to repress the promoter activities of poplar wood biosynthetic genes. We demonstrated that PtoMYB156 functions as a transcriptional repressor and negatively regulates secondary cell wall formation in poplar.

## Materials and Methods

### Plant materials and growth conditions

*Populus tomentosa* Carr. (Clone 741) was grown in a greenhouse under a 14-/10-h light/dark cycle with supplemental light (4500 lux) and at 23–25 °C. For gene expression pattern analysis in different tissues, including leaves, roots, stems, bark, xylem and phloem, were collected from 6-month-old poplar plants. Samples were frozen immediately in liquid nitrogen and stored at −80 °C until RNA isolation.

*Nicotiana benthamiana* plants were grown in a greenhouse at 23 °C with 16/8 hrs of day night cycle.

### Gene cloning and phylogenetic analysis

Total RNA was isolated from *P. tomentosa* Carr. using the Plant Mini Kit (Qiagen, Germany). First-strand cDNAs were synthesized from 2 μg of total RNA in 20 μl of reaction mixture using the RT-AMV transcriptase Kit (TaKaRa, Dalian, China). The coding sequence (CDS) of *PtoMYB156* was amplified by gene-specific primers (Supplementary Table S1). Thermal cycler programmes were as follows: 96 °C for 3 min followed by 32 cycles of 94 °C for 30 s, 56 °C for 30 s and 72 °C for 50 s, and a final extension step at 72 °C for 10 min. The amplification products were cloned into the plant binary vector pCXSN as previously described^35^.

The amino acid sequences of R2R3 MYB transcription factors in other species were obtained by BLAST searchers (http://www.phytozome.com). The deduced amino acid sequences were aligned with the program DNAMAN7.0 (Lynnon Corporation, USA). Phylogenetic analysis based on amino acid sequences was preformed using the Neighbor-Joining (NJ) method through MAGE 5.0^36^.

### Semi-quantitative RT-PCR and quantitative real-time PCR

Total RNA was extracted from different tissues of poplar plants using the Trizol Reagent (Tiangen, China). The gene-specific primers are listed in Supplementary Table S1. Semi-quantitative reverse transcription (RT-PCR) conditions were an initial denaturation step at 94 °C for 3 min, 28 cycles of 94 °C for 30 s, 58 °C, and 72 °C for 1 min, and an extension step at 72 °C for 10 min. The amplification products were resolved by 1% (w/v) agarose gel electrophoresis and visualized with ethidium bromide under UV light. A TP800 Real-Time PCR machine (TaKaRa, Japan) was used for quantitative real-time reverse transcription-PCR (qRT-PCR) analysis. The poplar *Ptr18S* gene was used as internal references to normalize the expression data. Three biological and three technical replicates were performed for each gene.

### Transformation of poplar

The *35S:PtoMYB156* construct was transformed into *Argobacterium tumefaciens* strain EHA105 using the freeze-thaw method. *Populus* transformation was performed according to the *Agrobacterium*-mediated leaf disc method previously established in our laboratory^37^. Putative transgenic plants were selected on woody plant medium (WPM)^38^ supplemented with 9 mg l^−1^ hygromycin. Transformed plants were sub-cultured by cutting shoot apices to WPM medium with 9 mg l^−1^ hygromycin. Rooted plantlets were acclimatized in pots at 25 °C in a 14-/10-h light/dark cycle and then transferred to the greenhouse for further studies.

### Subcellular localization of PtoMYB156

The *PtoMYB156* coding sequence was amplified from *P. tomentosa* Carr. with gene-specific primers (Supplementary Table S1) and ligated into the pCX-DG vector^35^ to produce a *35S-PtoMYB156:GFP* construct. The recombinant expression vectors were introduced into tobacco BY-2 cells by transient *Agrobacterium*-mediated transformation method^39^. The tobacco BY-2 cells were stained with 4′,6-diamidino-2-phenylindole (DAPI), and then photographed under a fluorescent microscope (Olympus BX53, Japan).

### Transcriptional repression in yeast

The cDNA encoding *PtoMYB156* was amplified by PCR and cloned into *Eco*RI and *Nco*I sites of pGBKT7 vector (Clontech). The VP16 motif was linked into pGBKT7 vector and fused with the C-terminal of PtoMYB156 protein. To determine the transcription activity of PtoMYB156, the GAL4BD/UAS/LacZ transcient assays were performed in yeast cells as described previously^40^. β-galactosidase assays were performed as described in the yeast protocols handbook (Clontech).

### Transient expression assay

The promoter fragments of secondary wall biosynthetic genes (*PtrCES17, PtrC4H2* and *PtrGT43B*) were amplified by PCR with gene-specific primers listed in Supplementary Table S1. These amplified fragments were fused to the *GUS* reporter gene in the modified pCambia1305.1 vector to generate reporter constructs, respectively. The *35S-PtoMYB156* construct were used as an effector. Tabacco leaves were infiltrated by *Argrobacterium* cells containing the effector and reporter with the agroinfiltration method^41^. After 3d of infiltration, GUS activity was quantitatively measured by spectrophotometry^42^.

### Histochemistry and microscopy

The free-hand cross-sections of fresh stems (6th internode) from 4-month-old plants were stained with 5% (w/v) phloroglucinol-HCl for lignin detection. Different tissues were fixed in formaldehyde acetic acid solution [formaldehyde:glacial acetic acid:ethanol (1:1:18)], dehydrated in graded ethanol series and embedded into paraffin. Sections (10 μm thickness) were cut with a razor blade and an Ultra-Thin Semiautomatic Microtome (FINESSE 325, Thermo). After the removal of paraffin, the samples were stained with 0.05% (w/v) toluidine blue or 5% (w/v) phloroglucinol-HCl, and observed under a light microscopy^43^. Cell wall thicknesses of fibers and vessels were measured using IMAGE-PRO PLUS software (MediaCybernetics, Bethesda, MD, USA). In all cases, pairs of similar cell types were selected for measurement.

### Measurement of total phenolics, flavonols, anthocyanins and proanthocyanidins

Quantitative determination of total phenolics was performed as described previously^25^. Total phenolic content was examined from standard curves obtained using dilutions of gallic acid, rutin and cyanidin chloride at 280, 360 and 520 nm, respectively.

Total flavonol content in poplar plants was measured according to the modified method reported previously^44^. Plant tissues (100 mg) were extracted in 3 ml of 80% methanol at 4 °C for 2 h. After centrifugation, aliquots of supernatant were dried under nitrogen and dried samples were incubated with 3 ml of 1N HCl at 90 °C for 2 h and extracted twice with 3 ml of ethyl acetate. Ethyl acetate extracts were pooled, dried under nitrogen, and resuspended in 200 μl of methanol. The absorbance at 415 nm was recorded on a spectrophotometer. The standard curve was prepared using 0, 50, 100, 150, 200, 250 mg/l of naringenin in methanol solutions. Flavonol content was calculated on the basis of naringenin level.

Total anthocyanin content of poplar plants was determined as described previously^44^. Briefly, 0.5 g of plant tissue were ground in liquid N_2_ and incubated in 5 ml of methanol: 0.1% HCl at 4 °C for 1 h, followed by shaking overnight at 120 rpm. After centrifugation (2,500 g, 10 min, 4 °C), 1 ml of water was added to 1 ml of extract, followed by addition of 1 ml of chloroform to remove chlorophyll. The absorbance of the supernatants was measured at 530 nm. Total anthocyanin concentration was calculated using the molar absorbance of cyanidin-3-O-glucoside.

For extraction of proanthocyanidins (PAs), poplar leaves were ground in liquid N_2_ and 0.5 g batches were extracted with 2.5 ml extraction solution (70% acetone and 0.5% acetic acid) by vortexing followed by sonication at 30 °C for 30 min. After centrifugation (2500 g, 10 min), residues were re-extracted twice. The supernatants were then extracted with 2 ml chloroform. The aqueous supernatant was extracted twice with chloroform and then three times with hexane. Samples were freeze dried, resuspended in extraction solution. Soluble PA content was determined using dimethylaminocinnamaldehyde (DMACA) reagent with catechin standards. Three independent experiments were performed for each sample.

### Lignin extraction and analysis

Internode samples (9^th^–15^th^ internodes) were harvested from the wild-type control and transgenic lines. Lignin analyses were carried out on dry extract-free cell wall residues, and ground to pass through a 40 mesh sieve, before extracted with benzene-ethanol (2:1, v/v) in a Soxhlet for 4 h, and then air-dried in a hood for several days until constant weight was achieved. Klason lignin content was determined in pre-extracted tissues as previously described^45^. The pretreatment of lignin monomer determination was as described previously^45^. After filtered with a membrane filter (0.22 μm), the final solution was prepared for HPLC analysis. Aliquots (20 μL) of the solution was injected into the Shimadzu HPLC system (Kyoto, Japan), equipped with a model LC-20AD binary gradient pump, an SPD-M20A diode-array detector (set at 280 nm), a SiL-20A auto sampler, a DGU-20A3 degasser and CTO-20A column oven. The analyses were performed by a inertsil ODS-SP column (4.6 × 250 mm, 5 μm) with CH_3_OH:H_2_O:HAc (25:74:1, v/v/v) carrier liquid (flow rate: 1.1 ml min^−1^)^46^,^47^.

### CRISPR/Cas9-mediated *PtoMYB156*-knockout in poplar

To construct the CRISPR/Cas9 gene knockout vector, the binary pYLCRIPSR/Cas9 multiplex genome targeting vector system^48^ was used as described by Fan *et al*.^49^. The *PtoMYB156* coding sequence was screened in the online tool ZiFiT Targeter Version 4.2 (http://zifit.partners.org/ZiFiT/Introduction.aspx)^50^. Three of putative target sites located at the first exon of the *PtoMYB156* coding sequence were chosen for designing the sgRNA sequences based on their GC content. Three pairs of oligos (Supplementary Table S1) were designed to specifically target *PtoMYB156* and sgRNA cassettes driven by the promoters of *Arabidopsis AtU3b, AtU6–1* and *AtU6-29*, respectively, were assembled into the binary CRISPR/Cas9 vector based on Golden Gate Cloning^51^.

Transgenic poplar plants were generated by *Agrobacterium*-mediated transformation as described previously^37^. For CRISPR/Cas9-based knockout of *PtoMYB156* in transgenic T_0_ poplar plants, the genomic DNA was isolated with a typical CTAB method, followed by PCR amplification and DNA sequencing. To further validate the targeted DNA insertions or deletions, the PCR product was cloned into the pMD19-T Simple vector (Takara, Dalian, China) and at least 15 clones for each transgenic line were selected for sequencing.

### Statistical analysis

The experimental data referred to plant height, internode length, biomass, cell wall thickness, lignin content, quantitative RT-PCR and GUS activity assays were subjected to statistical analysis using the Student’s *t* test program (http://www.graphpad.com/quickcalcs/ttest1.cfm). Quantitative difference between two groups of data for comparison in each experiment was found to be statistically significant (*P < 0.05; **P < 0.01).

### GenBank accession numbers for genes used in this study

The accession number of *PtoMYB156* in the GenBank database is KT990214. Other GenBank accession numbers for genes used in this study are as follows: EjMYB2 (KF767454), AmMYB308 (P81393), PdMYB221 (POPTR_0004s18020), EgMYB1 (CAE09058.1), LlMYB1 (GU901209), PvMYB4a (JF299185), TaMYB4 (JF746995), ZmMYB42 (NP_001106009), AtMYB4 (AAS10085.1), AtMYB3 (AT1G22640.1), AmMYB330 (P81395.1), PtrMYB182 (XP_002305872), GbMYBF2 (JQ068807), EJMYB1 (KF767453.1), AtMYB63 (AT1G79180), AtMYB58 (AT1G16490), EgMYB2 (AJ576023), PtrMYB3 (XM_002299908), PtrCESA2B (JX552008.1), GhMYBL1 (KF430216), ZmMYB31 (NP_001105949), PtrMYB20 (XM_002313267), PtrCCOAOMT1 (Potri.009G099800.4), PtrCCR2 (Potri.003G181400.2), PtrCOMT2 (Potri.012G006400.2), PtrCAD1 (Potri.009G095800.2), PtrC3H3 (Potri.006G033300.2), PtrPAL4 (Potri.006G126800.1), PtrHCT1 (Potri.001G042900.2), PtrC4H2 (Potri.001G042900.2), Ptr4CL5 (Potri.003G188500.2), PtrF5H2 (Potri.007G016400.1), PtrCESA17 (Potri.002G257900) and PtrGT43B (Potri.016G086400.1).

## Results

### Isolation and characterization of PtoMYB156

**Figure 1:**
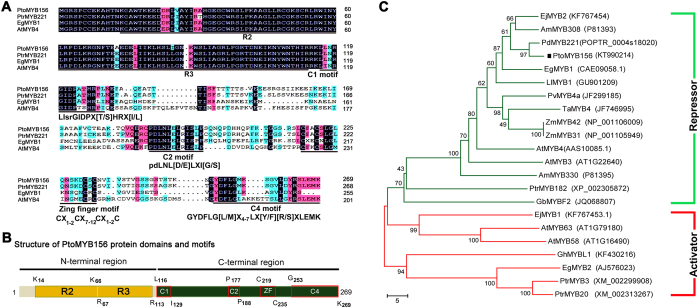
Comparison of PtoMYB156 with other R2R3 MYB amino acid sequences. (**A**) Multiple sequence alignment between PtoMYB156 and the other R2R3-MYB subfamily 4 proteins. Identical amino acids are shaded in gray. The potential functional motifs and conserved MYB domain are underlined. (**B**) Structure of PtoMYB156 protein domains and potential motifs. The boxed sequences are C1, C2, Zf and C4 motifs. (**C**) Phylogenetic analysis of PtoMYB156 and other R2R3-MYB proteins by the neighbor-joining method using MEGA version 5.0. The number beside the branches represents bootstrap value based on 1,000 replications. The scale bar represents 5 substitutions per site.

A putative R2R3 MYB transcription factor gene was obtained by BLAST search in the poplar database using AtMYB4 as a query sequence. The full-length open reading frame (ORF) was amplified by RT-PCR from cDNA of leaves of 6-month-old *P. tomentosa*. The sequence, named PtoMYB156 (accession no. KT990214), encodes a protein of 269 amino acid resides (Fig. 1A) with a predicted molecular mass of 30 kD and a calculated pI of 8.5. The sequence alignment of PtoMYB156 with other MYB repressors showed that PtoMYB156 has a highly conserved R2-R3 domain at the N-terminal region and the C-terminal domain is more divergent (Fig. 1A). Some typical protein motifs of were found at the C-terminal of the MYB subgroup 4 transcription factors^24^,^26^,^27^,^52^. These motifs, including the C1 (LlsrGIDPX[T⁄S]HRX[I/L]), C2 (pdLNL[D⁄E]LXI[G/S]), C4 (GYDFLGLX_4–7_LX[Y/F][R/S]XLEMK) and ZF (CX_1–2_CX_7–12_CX_1–2_C) motifs were found in the C-teriminal of PtoMYB156 protein (Fig. 1B).

A phylogenetic tree was constructed using the neighbor-joining method with the protein sequences of PtoMYB156 and other MYB factors involved in the regulation of the phenylpropanoid pathways (Fig. 1C). Phylogenetic analysis showed that PtoMYB156 is more closely related to AmMYB308^53^, EjMYB2^54^, EgMYB1^27^ than the phenylpropanoid/lignin biosynthesis repressors such as AtMYB4^22^, AmMYB308^55^, ZmMYB42^25^, ZmMYB31^24^. In addition, PtoMYB156 shares a high level of amino acid sequence identity with PdMYB221 from *P. deltoids*^34^, indicating that they are homologous genes and have the similar biological functions as defined in regulating secondary wall biosynthesis in different species.

### Expression patterns of *PtoMYB156*

To determine the tissue-specific expression profiles of *PtoMYB156* in poplar, we extacted total RNA from different tissues and performed qRT-PCR analysis. The *PtoMYB156* was expressed in all the tissues tested, with the highest expression in old leaves and lowest expression in roots (Supplementary Fig. S1). Transcript accumulation of *PtoMYB156* was also detected throughout the stem, including xylem, phloem and bark. On the other hand, in transgenic Arabidopsis plants harboring the *GUS* (β-glucuronidase) gene driven by the promoter of *PtoMYB156*, histochemical GUS staining showed that GUS activity was detected in all tissues of transgenic plants, especially in vascular tissues of roots, stems and leaf veins (Supplementary Fig. S2).

### PtoMYB156 is a transcriptional repressor localized to the nucleus

**Figure 2:**
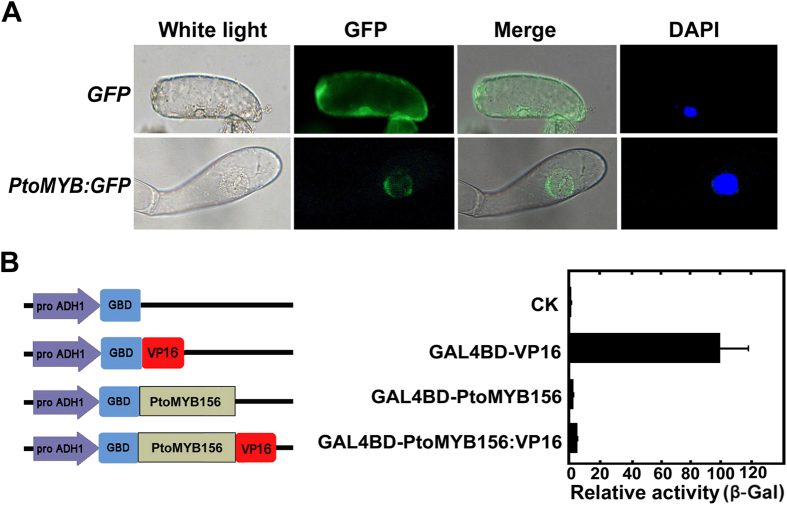
Subcellular localization and transactivation assays of PtoMYB156. (**A**) Transient expression of 35S-PtoMYB156:GFP fusion proteins in tobacco BY-2 cells. The position of nucleus was ensured by DAPI staining. A tobacco BY-2 cell expressing PtoMYB156:GFP or GFP alone shows its localization in the nucleus or in the cytoplasm, respectively. (**B**) Transcriptional activation analysis of PtoMYB156 analyzed by the chimeric reporter/effector assay in yeast. GBD, GAL4 DNA binding domain; VP16, activation motif of the VP16 protein; GALBs, GAL4 protein binding sites. Data represent mean ± SD from three biological replicates. GAL4DB null vector was used as a negative control and GAL4BD fused with VP16 was used a positive control.

To test whether PtoMYB156 is localized to the nucleus, the open reading frame (ORF) of PtoMYB156 was fused into the C-terminal of the *GFP* gene of a ZeBaTA vector pCXDG^35^ under the control of the *CaMV 35S* promoter. The construct with a PtoMYB156:GFP fusion protein was transformed into protoplasts from tobacco BY-2 cells. As shown in Fig. 2A, GFP fluorescence in cells with *PtoMYB156:GFP* was shown to localize to the nucleus by confocal microscopy, whereas GFP alone was distributed throughout the entire cells.

To determine transcriptional activity of PtoMYB156, the ORF of PtoMYB156 was fused with the VP16 activation motif from herpes simplex virus protein VIP16^56^ and GAL4 binding domain (Fig. 2B). The reporter construct contained the *LacZ* reporter gene driven by the pADH1 promoter with GAL4 binding motif. After expression of reporter and effector constructs in yeast, β-galactosidase assays showed that the transcriptional activation activity of VP16 domain was reduced markedly when fused to PtoMYB156 protein, indicating that PtoMYB156 has transcriptional repression activities (Fig. 2B).

### Ectopic expression of *PtoMYB156* represses phenylpropanoid biosynthesis in poplar

**Figure 3:**
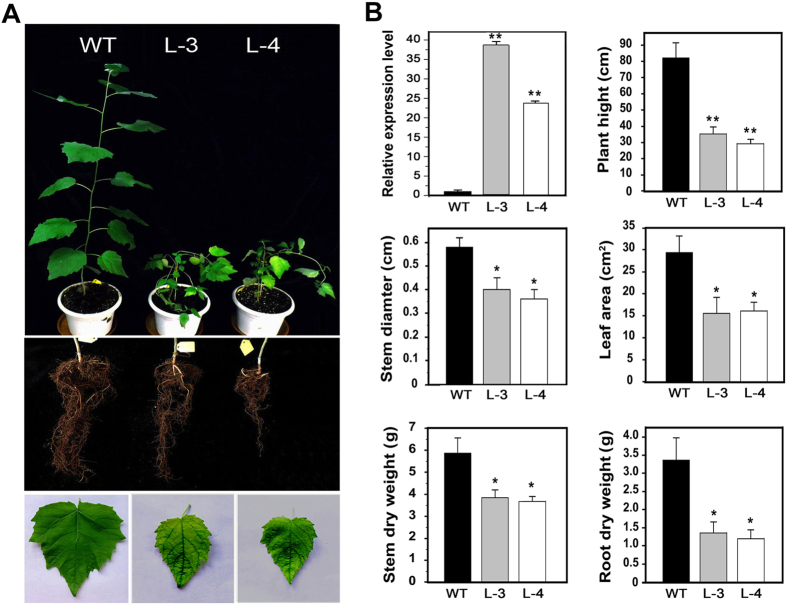
Phenotype of transgenic poplar overexpressing *PtoMYB156*. (**A**) Four-month-old poplar plants grown in the greenhouse. Overexpression of *PtoMYB156* caused retarded growth in transgenic plants compared with the wild-type control. WT, wild type plants; L3 and L4, transgenic lines 3 and 4. (**B**) qRT-PCR analysis of the expression of *PtoMYB156* in transgenic plants overexpressing *PtoMYB156,* and plant height, stem diameter, leaf area and biomass of stems and roots from the control and transgenic plants. Data are means ± SE (n = 10). Student’s *t* test: *P < 0.05; **P < 0.001.

In order to establish the biological function of *PtoMYB156*, we overexpressed it under the control of the *CaMV* promoter in Chinese white poplar (*P. tomentosa* Carr.). A few independent transgenic lines, such as line 3 (L3) and line 4 (L4), showed high mRNA levels of *PtoMYB156* (Fig. 3A). Compared with wild-type plants, transgenic lines overexpressing *PtoMYB156* displayed pleiotropic phenotypes such as decreased plant height, thinner stems, smaller leaves and fewer roots (Fig. 3B). After growth for 4 months in a greenhouse with a 14-/10-h light/dark cycle, the transgenic lines with severe reduction in height were 56–63% shorter and had a reduced diameter of 30–36% than the controls, respectively (Fig. 3B). In addition, there were significant differences in biomass of shoots and roots between transgenic and control plants when dry weight was measured (Fig. 3B).

**Figure 4:**
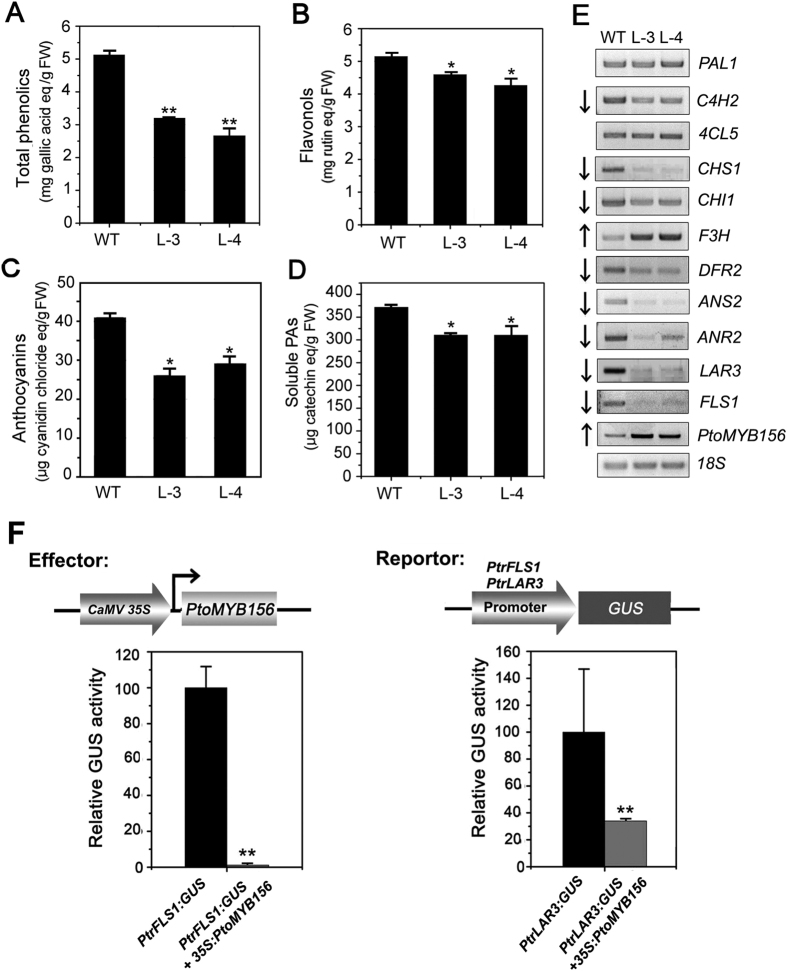
Constitutive expression of *PtoMYB156* in poplar repressed the phenylpropanoid biosynthetic genes and reduced the accumulation of phenylpropanoid compounds. (**A–D**) Quantification of different phenylpropanoid compounds, including total phenolics (**A**), flavonols (**B**), anthocyanins (**C**) and soluble PAs (**D**), in transgenic plants overexpressing *PtoMYB156* and the control (wild-type). (**E**) Transcript levels of phenylpropanoid biosynthetic genes were detected by semi-quantitative RT-PCR in two *35S:PtoMYB156* independent lines (L3 and L4) and compared with wild-type lines. *18S* was used as a quantitative control. (**F**) PtoMYB156 activates promoters of flavonoid biosynthetic genes. The vectors containing *PtoMYB156* and the promoters of flavonoid biosynthetic genes used for transfection of tobacco leaves are indicated. Each column represents the mean value of three independent experiments with error bars indicating ± ses. Student’s *t* test: *P < 0.05; **P < 0.001.

Since PtoMYB156 shares significant similarity with other phenylpropanoid/lignin biosynthesis repressors such as AtMYB4^22^, AmMYB308^55^, ZmMYB42^25^, ZmMYB31^24^ and ZmMYB11^19^, we investigated whether PtoMYB156 could also negatively regulate the biosynthesis of phenylpropanoid compounds in transgenic plants. Quantification analysis showed a strong reduction in accumulation of total phenolics, flavonols, anthocyanins and soluble PAs in *35S:PtoMYB156* lines compared with the wild type (Fig. 4A–D). RT-PCR analysis for two independent lines indicated that *PtoMYB156* can act as a repressor of expression of phenylpropanoid structural genes in transgenic plants (Fig. 4E). The expression of genes involved in the flavonoid biosynthetic pathway, including *CHS1, CHI1, DFR2, ANS2, ANR2, FLS1* and *LAR3*, appeared strongly down-regulated in *35S:PtoMYB156* lines compared to wild-type plants. Additionally, the expression of F3H was clearly up-regulated compared to the wild type (Fig. 4E).

To further investigate which structural genes of the flavonoid biosynthetic pathway were repressed by PtoMYB156, we established a transient expression method using tobacco leaves by *Agraobacterium*-medeated transformation. In the effector plasmid, *PtoMYB156* was driven by the *Cauliflower mosaic virus* (CaMV) 35S promoter. The promoters of *PtrFLS1* and *PtrLAR3* were used to control the expression of the GUS reporter gene. PtoMYB156 strongly suppressed the promoters of the gene *PtrFLS1* (reduced to approximately 5%) and *PtrLAR3* (reduced to approximately 36%) (Fig. 4F), indicating that it can repress the different flavonoid pathways.

### Overexpression of *PtoMYB156* affects secondary cell wall development in transgenic poplar

**Figure 5:**
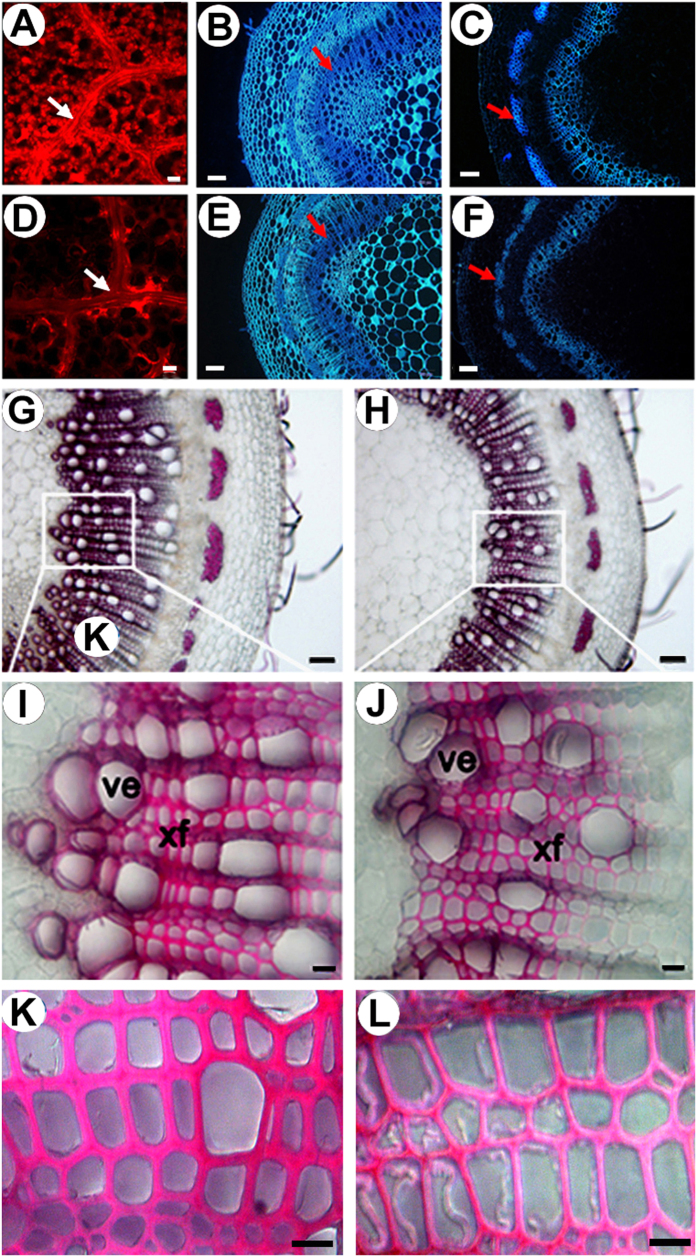
Microscopic analysis of leaves and stems from wild-type and transgenic *35S:PtoMYB156* plants. Compared with a wild-type leaf (**A**) and stem (**C**), lignin auto-fluorescence images of the *PtoMYB156*-overexpression plants showed the less lignified secondary wall thickening in leaf veins (**D**) and stem cross-sections (**F**). Calcofluor white staining of stem cross-sections showed an reduction in cellulose content in transgenic *35S:PtoMYB156* lines (**E**), compared with the control (**B**). (**G–J**) General view of stem vascular tissues stained by phloroglucinol-HCl in basal transverse sections of stems from wild-type (**G,I** and **K**) and transgenic lines overexpressing *PtoMYB156* (**H**,**J** and **L**). Xf, xylary fibers; ve, vessel; pf, phloem fibers. Scale bars: 100 μm in (**B,C,E,F,G,H**); 5 μm in K, L; 20 μm in (**A,D,I,J**).

To evaluate whether PtoMYB156 affects lignin biosynthesis in poplar, stem cross-sections were observed under UV light. Confocal microscopy of lignin autofluorensence showed that lignified secondary wall thickening was mainly observed in veins of wild-type leaves (Fig. 5A), but weaker signals in transgenic plants (Fig. 5D). Consistently, the less intense autofluorescence of lignin and cellulose was detected in the stem cross-sections of transgenic plants overexpressing *PtoMYB156* (Fig. 5E and F) compared with the wild type (Fig. 5B and C). Phloroglucinol-HCl staining of lignin in stem cross-sections revealed that the typical intense red stain of secondary cell walls in wild-type plants (Fig. 5G), but less intense staining was detected in transgenic 35S:*PtoMYB156* plants (Fig. 5H). Compared with the control (Fig. 5I), secondary xylem tissue of transgenic plants (Fig. 5J) was substantially reduced. Quantitative determinations showed that, on average, cell wall thickness was reduced by about 13% and 28% for xylem vessel cells and xylem fiber cells (Supplementary Table S2), respectively. Toluidine blue-*O* staining of stem cross-sections indicated that mean cell area of xylem and phloem fibers of *35S*:*PtoMYB156* plants was significantly reduced compared with the control plants (Fig. S3, Supplementary Table S3).

In order to quantify lignin modifications, we measured Klason lignin content in the stems of wild-type and transgenic plants (Supplementary Table S4). The results showed that lignin accumulation was significantly decreased (about 14.3%) in stems of 4-month-old poplar plants. But the lignin monomer yield and composition (S:G ratio) was not significantly changed (Supplementary Table S4).

### Overexpression of *PtoMYB156* affects the expression of secondary wall biosynthetic genes in transgenic poplar

**Figure 6:**
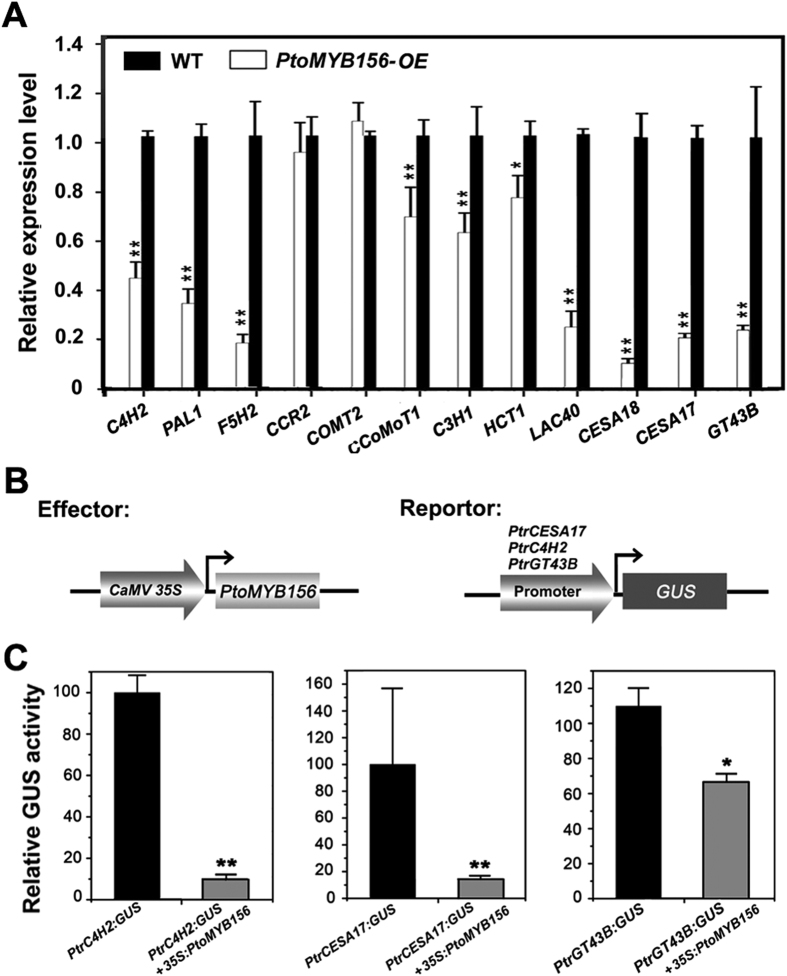
PtoMYB156 repressed the expression of the secondary wall biosynthetic genes. (**A**) Gene expression analysis of these genes associated with secondary wall biosythesis of wild-type and *PtoMYB156*-overexpression plants. Transcript accumulation of genes involved in secondary cell wall formation in poplar, including *PtoCCOAOMT1, PtoCCR2, PtoCOMT2, PtoC3H3, PtoHCT1, PtoLAC40, PtoC4H2, PtoPAL1, PtoCESA18, PtoCESA17*, and *PtoGT43B*, was quantified by qRT-PCR. The reference gene *18S* rRNA was used as an internal control. The expression level of each gene in the wild type was set to 1. Error bars represent ± SD of three biological replicates. Student’s *t* test: *P < 0.05; **P < 0.01. (**B**) Diagrams of the effector and reporter constructs used for transcriptional activity analysis. (**C**) Transcriptional activity analysis showed that PtoMYB156 repressed the expression of the GUS reporter gene driven by the *PtrC4H2, PtrCESA17*, and *PtrGT43B* promoters. GUS expression in tobacco leaves transfected with the reporter construct alone was used as a control. Error bars represent ± SEs of three biological replicates. Student’s *t* test: *P < 0.05; **P < 0.01.

Quantitative RT-PCR analysis with gene-specific primers (Supplementary Table S1) was used to determine expression levels of the genes encoding the enzymes of secondary wall biosynthesis. In transgenic *35S*:*PtoMYB156* lines, the expression of these genes involved in the biosynthesis of wood components, including cellulose (*CES17/18*), xylan (*GT43B*) and lignin (*F5H2, CCoMOT1, C3H1, HCT1, LAC40, C4H2*), was significantly downregulated, compared with the control (Fig. 6A). Overexpression of *PtoMYB156* in poplar also resulted in downregulation of *PtoPAL1* involved in phenylpropanoid pathway. In addition, the expression of several secondary wall-associated transcription factors, PtoMYB003/018/020/021/028/152, were repressed, while PtoKNAT7, a transcriptional repressor^57^, was induced in *35S*:*PtoMYB156* lines (Supplementary Fig. S4). These results indicate that PtoMYB156 could function as a negative regulator of secondary wall biosynthesis in poplar.

### The *PtrCESA17, PtrC4H2* and *PtrGT43B* promoters are repressed by PtoMYB156

To investigate the roles of PtoMYB156 in the regulation of secondary wall biosynthesis, we determined whether it was capable of repressing the promoters of poplar wood biosynthetic genes. The *PtrCESA17, PtrC4H2 and PtrGT43B* promoters were amplified from the genomic DNA of *P. trichocarpa* and fused to the GUS reporter gene. The reporter and effector constructs (Fig. 6B) were co-transfected into *Arabidopsis* leaves by *Agrobacterium*-mediated method. GUS activity assays showed that PtoMYB156 was able to significantly repress expression of the GUS reporter gene under the control of the *PtrCESA17, PtrC4H2 and PtrGT43B* promoters (Fig. 6C).

### Knockout of the *PtoMYB156* gene affected secondary wall formation in poplar

**Figure 7:**
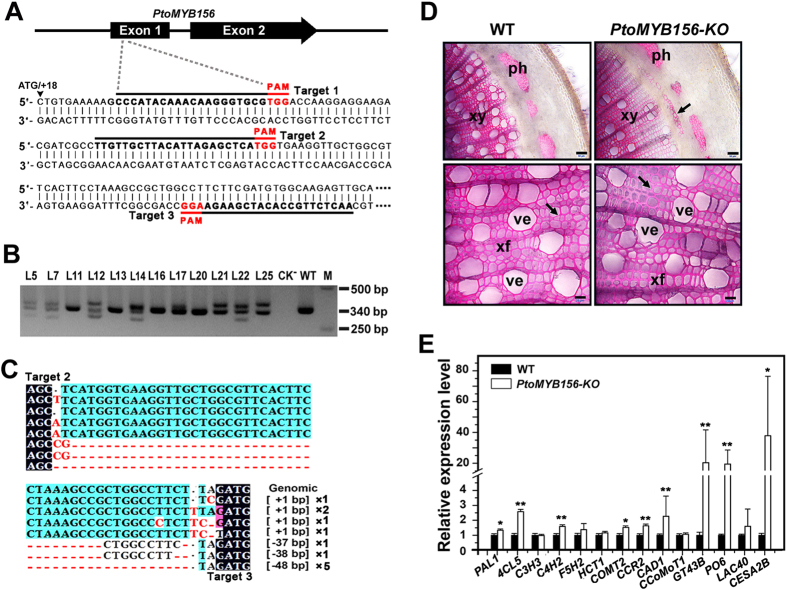
*PtoMYB156* was knockouted by the CRISPR/Cas9-mediated targeted mutagenesis in the first generation of transgenic poplar plants. (**A**) DNA sequences at the sgRNA target site within the encoding sequence of the *PtoMYB156* gene. The PAM sequence is shown in red and targeted sequences are underlined. (**B**) PCR analysis of total DNA extracts from independent transgenic T0 poplar plants showing the mutations of *PtoMYB156* by the the CRISPR/Cas9 system. CK-, negative control (without DNA template): WT, wild type; M, DNA marker. (**C**) Confirmation by DNA sequencing of Cas9/sgRNA-mediated mutagenesis of the sgRNA target sites within the *PtoMYB156* gene. Twenty-five cloned DNA fragments from PCR amplified sgRNA target regions of *PtoMYB156* from three independent transgenic lines (L5, L7 and L12) were subjected to DNA sequencing. Deleted nucleotides are depicted as red dots and inserted nucleotides are shown in red. The nucleotide length of insertions and/or deletions (In/Del) are presented in the column to the right. (**D**) *PtoMYB156* knockout induced ectopic deckout line stained for lignin with phloroglucinol HCl. Scale bars: 50 μm (top); 20 μm (bottom). (**E**) Gene expression analysis of secondary wall biosynthetic genes in *PtoMYB156* knockout plants. The reference gene *18S* rRNA was used as an internal control. The expression level of each gene in the wild type was set to 1. Error bars represent ± SEs of three biological replicates. Student’s *t* test: *P < 0.05; **P < 0.01.

To further analyze its genetic function, we used a CRISPR/Cas9-based reverse genetic system^49^ to knock-out the *PtoMYB156* gene in the *P. tomentosa* genetic background. Three 20-bp sequences with tandem guanosine nucleotides (PAM) at the first exon region of *PtoMYB156* were chosen as sgRNA complementary sites (Fig. 7A). The binary vector with the CRISPR/Cas9 system was introduced into poplar by *Agrobacterium*-mediated transformation and 12 independent transgenic lines were generated. The integration of the transgenes into the genome of transgenic plants was verified by PCR with gene-specific primers for the hygromycin phosphotransferase (Hyg) gene (Supplementary Fig. S5). To detect mutations in the target region, amplified polymorphic sequence analysis was conducted using genomic DNA extracted from independent transgenic lines. At least three PCR products with different size were obtained from transgenic lines when amplified with gene-specific primers for *PtoMYB156* (Fig. 7B). DNA sequencing analysis was performed on cloned-PCR products from three *PtoMYB156* knock-out (*PtoMYB156-KO*) lines (L5, L7 and L12). We found that 48% (12/25) of cloned-PCR products contained mutations in *PtoMYB156-KO* line 5 (Fig. 7C). Small deletions were commonly found at the site of cleavage, whereas large deletions (>30 bp) were also observed at a low frequency.

No phenotypic alterations were observed in *PtoMYB156-KO* lines compared with the wild-type (Supplementary Fig. S6). However, when stem cross-sections were examined, ectopic deposition of lignin was detected in some secondary phloem cells of *PtoMYB156-KO* plants (Fig. 7D). Histochemical analyses showed that secondary cell walls of stem vascular tissue were obviously thicker in *PtoMYB156-KO* lines compared with the control (Fig. 7D). Quantitative RT-PCR analysis revealed that knockout of *PtoMYB156* resulted in an increase in expression of secondary wall biosynthetic genes for lignin (*PAL1, 4CL5, C4H2, COMT2, CCR2, CAD1*), xylan (*GT43B*) and cellulose (*CESA2B*) (Fig. 7E). These results indicated that *PtoMYB156* knockout triggered the expression of the genes involved in secondary wall formation and lignin biosynthesis.

## Discussion

In tree species, a number of wood-associated transcription factors, including NAC and MYB proteins, have been demonstrated to be involved in the coordinated regulation of secondary wall biosynthesis during wood formation^4^,^14^,^15^,^16^,^27^,^32^,^58^,^59^. In this study, we characterize a poplar repressor-like R2R3-MYB, PtoMYB156, and demonstrated that it was not only able to down-regulate different branches of the phenylpropanoid pathway but also negatively control secondary cell wall formation. Protein sequence analysis revealed PtoMYB156 contains four conserved motifs, C1, C2, Zf and C4, in the C-terminal region (Fig. 1B). In previous studies, C2 and C4 motifs have been demonstrated to function as a repression motif^22^,^24^,^27^. Phylogenetic tree analysis showed that PtoMYB156 is an ortholog of *Eucalyptus* EgMYB1, which acts as a transcriptional repressor of lignin biosynthesis^27^,^60^, in poplar (Fig. 1C). Transcriptional activity assays showed that PtoMYB156 suppressed *LacZ* expression in yeast (Fig. 2B), suggesting that it is a transcriptional repressor. Overexpression of *PtoMYB156* repressed the expression of various phenylpropanoid structural genes (Fig. 4E), resulting in significant reduction in the amounts of total phenolic compounds, lignins, flavonols, anthocyanins and PAs (Fig. 4A–D). In a previous study, PdMYB221, the nearest homolog of PtoMYB156 (Fig. 1C), appears to be involved in the regulation of secondary wall biosynthesis during wood formation^34^, however, it is unclear whether this gene also can impact on the phenylpropanoid pathway.

Several MYB transcription factors, including MYB4/7/32 (Arabidopsis), ZmMYB31/42 (maize), PvMYB4 (switchgrass) have been characterized to be transcriptional repressors of lignin biosynthesis^22^,^23^,^24^,^25^,^26^. Overexpression of these genes resulted in down-regulating expression of lignin biosynthetic genes and a reduction in lignin content. In *Eucalyptus, EgMYB1* overexpression in poplar and *Arabidopsis* led to repressing expression of biosynthesis genes not only for lignin but also for cellulose and xylan. Thus, EgMYB1 was a negative regulator for the entire secondary wall biosynthesis^27^. More recently, PdMYB221, a paralogous gene of PdMYB156, from *P. deltoides* have been demonstrated that it is involved in the negative regulation of secondary wall formation through the direct and indirect repression of secondary wall biosynthetic gene expression in transgenic *Arabidopsis*^34^. In our study, overexpression of *PtoMYB156* in poplar resulted in a dramatic effect on growth and development by reducing shoot height, stem diameter and leaf size (Fig. 3).

Chemical staining and quantitative analysis of *PtoMYB156*-overexpressor plants showed that lignin content was significantly reduced (Fig. 5 and Supplementary Table S4) and secondary wall thickness of fibers and vessels was also markedly decreased (Fig. 5 and Supplementary Table S3), compared with the control. Interestingly, the amount of S and G subunits were reduced in *PtoMYB156* overexpressors but no significant effect on the S:G ratio (Supplementary Table S4). In agreement with these observations, quantatitive RT-PCR analysis showed that *PtoMYB156* overexpression repressed a number of lignin biosynthetic genes (*C3H1, CCoMOT1, HCT1, LAC40, C4H2* and *PAL1*), two *CESA* genes (*CESA17* and *CESA18*) and one xylan biosynthetic gene (*GT43B*) (Fig. 6A). These data indicated that PtoMYB156 acts as a negative regulator of secondary cell wall formation in poplar. However, the detailed functions of PtoMYB156 to regulate secondary cell wall biosynthesis in poplar remain largely unknown and need to be further explored in the future.

Although the secondary wall biosynthetic genes were repressed by PtoMYB156 in transgenic poplar, it is still unknown that this suppression is directly or indirectly regulated through the binding of PtoMYB156 to the AC elements of secondary wall biosynthetic gene promoters. Other MYB factors as repressors have been established to directly bind target gene promoters^22^,^24^,^26^,^34^,^61^. In *Arabidopsis*, for example, the R3 domain of AtMYB4 is necessary to bind DNA, because mutating this region impeded the binding ability of AtMYB4, resulting in abolishing its ability to repress transcription^22^. ZmMYB31 was able to bind directly to the promoters of the maize *ZmCOMT* and *ZmF5H* genes, resulting in the repression of lignin biosynthetic gene expression^24^. The poplar PdMYB221 functioned as a transcriptional repressor and directly suppressed the expression of secondary wall biosynthetic genes including *PdCESA8, PdGT47C* and *PdCOMT2*^34^. We found that AC-like elements are also present in the promoter regions of other structural genes of secondary wall biosynthetic pathway in poplar and promoter activity analysis in transient assays showed that PtoMYB156 repressed the promoters of poplar *PtrC4H2, PtrCEASA17* and *PtrGT43B* (Fig. 6C). However, direct evidence for binding of PtoMYB156 protein to the promoters of secondary wall biosynthetic genes is still lacking.

To further determine the function of PtoMYB156, we created transgenic poplar plants with knockout mutations in *PtoMYB156* using the CRISPR/Cas9 system (Fig. 7). As described previously^49^, three sgRNA target sites located in the first exon of the *PtoMYB156* gene were chosen as target sequences (Fig. 7A). Interestingly, most of the *PtoMYB156* mutant alleles were deletions and insertions at the second and third target sites and no mutation was found in the first target site (Fig. 7C), indicating that selection of the optimum target-sites for sgRNA is particularly important for effectively directing gene-specific editing in *Populus*. In our study, a big fragment deletion was also detected in several *PtoMYB156*-*KO* lines (Fig. 7B and C). Similar results were found in other plant species such as *Arabidopsis*^62^, tomato^63^ and rice^48^. *PtoMYB156-KO* plants showed normal phenotypes (Supplementary Fig. S6), but exhibited a ectopic deposition of lignin in phloem tissues and an increase in secondary wall thickening in xylem cells compared with the control (Fig. 7D). Quantatitive RT-PCR analysis showed that knockout of *PtoMYB156* in poplar resulted in an increase in the transcriptional levels of secondary wall-associated genes (Fig. 7E). Our results indicated that knockout of *PtoMYB156* in poplar may abolish the direct suppression of *PtoMYB156* in regulating secondary wall biosynthesis. In Arabidopsis, a knockout mutant of *AtMYB4* showed an increase in sinapate esters, resulting in more tolerant to UV-B and transcript level of the gene encoding C4H, which was a major target of AtMYB4, was upregulated^22^. The mutation of *AtMYB32*, a transcription factor closely related to *AtMYB4*, resulted in aberrant pollen and male sterility and increased expression of *COMT*^23^.

Taken together, this study provided the evidence for the biological functions of PtoMYB156 as a negative regulator of phenylpropanoid pathway and secondary wall formation during wood formation in poplar. Combined with this findings in our study and other studies of poplar transcription factors (NAC and MYB)^9^, we propose that secondary wall formation requires fine-tuning spatiotemporal regulation in poplar, and transcription activators or repressors could provide a mechanism to ensure tight regulation of secondary wall biosynthesis in vascular tissues of poplar.

## Additional Information

**How to cite this article**: Yang, L. *et al*. PtoMYB156 is involved in negative regulation of phenylpropanoid metabolism and secondary cell wall biosynthesis during wood formation in poplar. *Sci. Rep.* **7**, 41209; doi: 10.1038/srep41209 (2017).

**Publisher's note:** Springer Nature remains neutral with regard to jurisdictional claims in published maps and institutional affiliations.

## Supplementary Material

Supplementary Information
