# Supplementary material for: PtoMYB156 is involved in negative regulation of phenylpropanoid metabolism and secondary cell wall biosynthesis during wood formation in poplar
Source: Sci Rep. 2017 Jan 24;7:41209. doi: 10.1038/srep41209 (PMC5259741; doi:10.1038/srep41209)
Supplement: Supplementary Information [file srep41209-s1.pdf]

PtoMYB156 is involved in negative regulation of phenylpropanoid metabolism and secondary cell wall biosynthesis during wood formation in poplar

Authors: Li Yang, Xin Zhao, Lingyu Ran, Chaofeng Li, Di Fan, Keming Luo

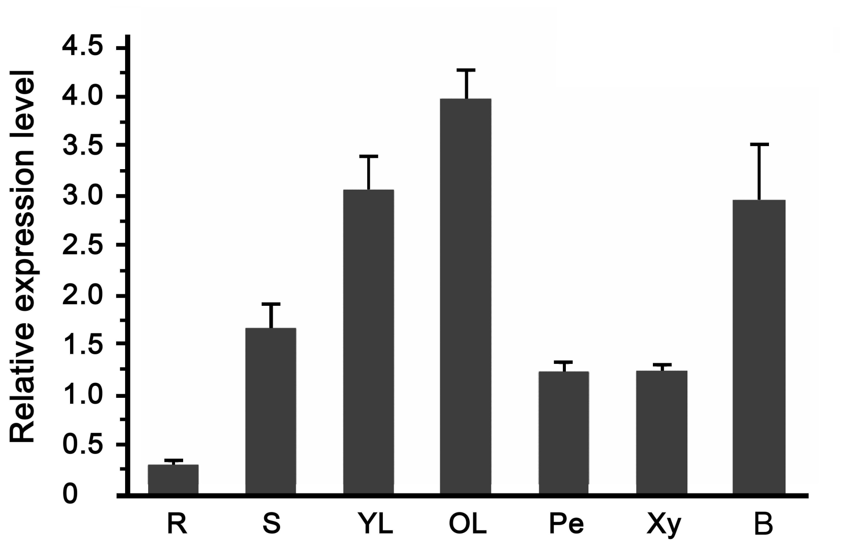

SupplementaryFig. S1 Expression patterns of *PtoMYB156* in different tissues of poplar

Quantitative real-time PCR analysis was performed to examine transcript levels of *PtoMYB156* in various tissues of *P. tomentosa* Carr. Total RNA was isolated from roots, stems, petioles, old leaves (the sixth leaf from the apex) and young leaves (the first leaf). The reference gene *18S* was used as the reference in different tissues. R, root; S, stem; YL, young leaf; OL, old leaf; Pe, petiole; Xy, xylem; B, bark. Error bars represent standard deviations (SD) of three biological replicates.

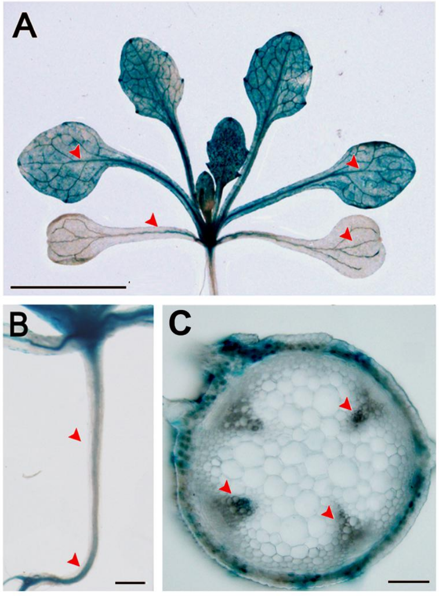

SupplementaryFig. S2. GUS expression of the *PtoMYB156* promoter in transgenic *Arabidopsis*. Seedlings (A), roots (B) and stems (C). Bars: 1 cm (A), 500 μm (B), 200 μm (C).

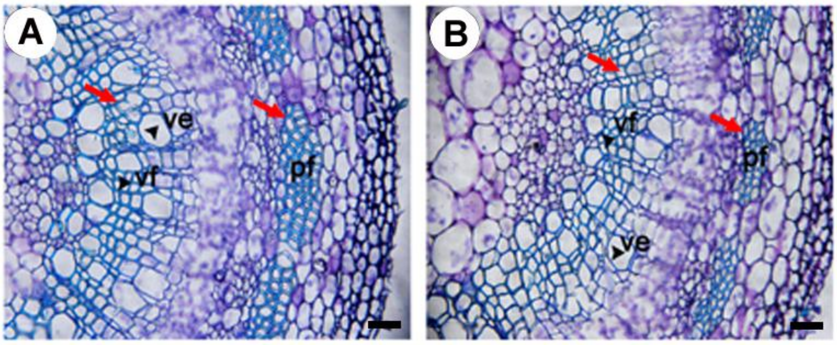

SupplementaryFig. S3. General view of stem vascular tissues stained by Toluidine blue-O in basal inflorescence stem transverse sections of wild-type (A) and transgenic lines overexpressing *PtoMYB156* (B) plants. Xf, xylary fibers; ve, vessel; pf, phloem fibers. Scale bars: 50 μm in K, L.

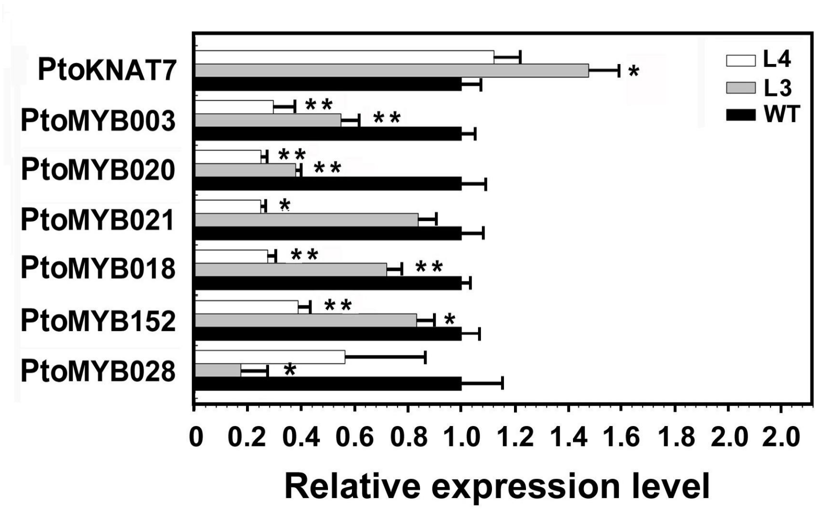

SupplementaryFig. S4. Quantatitive RT-PCR detecting the expression of several transcription factors associated with secondary wall biosynthesis in transgenic *35S:PtoMYB156* plants. The poplar 18S rRNA was used as an internal control. The expression level of each gene in the wild type was set to 1. Error bars represent SD of three biological replicates. Student's *t* test: *, $P < 0.05$; **, $P < 0.01$.

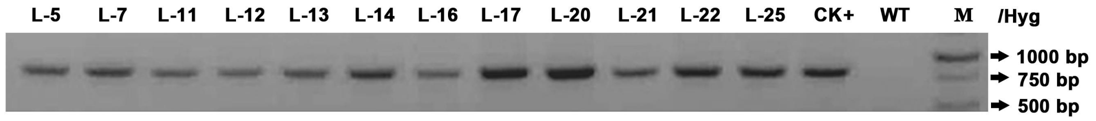

SupplementaryFig. S5. PCR analysis of transgenic poplar plants. Genomic DNAs were isolated from hygromycin-resistant plants transformed with the *35S:PtoMYB152* vector. M, DL2000 DNA Marker; CK+, corresponding plasmid DNA (positive control); WT, wild-type plants (negative control).

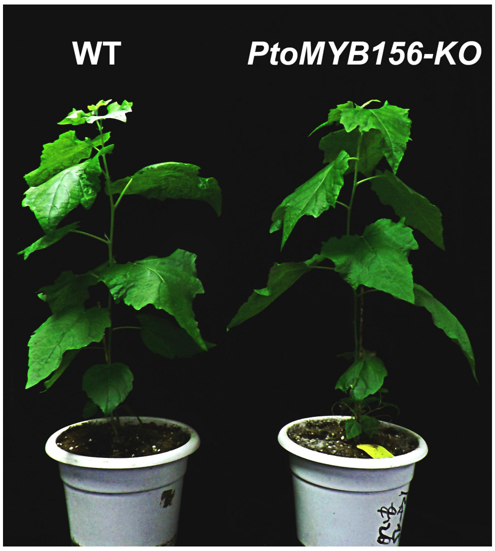

SupplementaryFig. S6. Phenotype of *PtoMYB156-knockout* transgenic poplar.

SupplementaryTable S1. Primers used in this study.

Primers	Sequence (5' -3')	Purpose
PtoMYB156-F	CCTCCGTCTCTAGTCCTACC	Primers for cloning
PtoMYB156-R	TCCCTTTCTCCTTTCTCTCC	
PtoMYB156-F	CCTCCGTCTCTAGTCCTACC	Primers for detecting mutation
PtoMYB156-Cas9-R	GGAGGCTATGGAGTTTGATA	
Hyg-F	CTTCTACACAGCCATCGGTCCAGA	
Hyg-R	GATGTAGGAGGGCGTGGATATGTC	
PtoMYB156-T1-F	ATTGCCCATACAAACAAGGGTGCG	Multiple assembled Cas9/sgRNA construct
PtoMYB156-T1-R	AAACCGCACCCTTGTTTGTATGGG	
PtoMYB156-T2-F	GTCAACTCTTGCCACATCTAAGA	
PtoMYB156-T2-R	AAACTCTTAGATGTGGCAAGAGT	
PtoMYB156-T3-F	ATTGTTGCTTACATTAGAGCTCA	
PtoMYB156-T3-R	AAACTGAGCTCTAATGTAAGCAA	
QPto18S-F	CGAAGACGATCAGATACCGTCCTA	quantitative PCR
QPto18S-R	TTTCTCATAAGGTGCTGGCGGAGT	
QPtoPAL1-F	CCATCCAGGTCAAATTGAGGCTGCT	

QPtoPAL1-R	ACTTCTTAGCTGCCTTCATGTAAGCT	
QPtoC4H2-F	GAAATGTGCAATTGATCATATTTG	
QPtoC4H2-R	ATTGCAGCAACATTGATGTTCTCC	
QPto4CL5-F	ATTCTGTGCGTGCTGCCTATGTTC	
QPto4CL5-R	AATTGCAGCACCAACTCTCAACCC	
QPtoHCT1-F	ATCAGCATGTAAGGCACGCGG	
QPtoHCT1-R	TGCCAAAGTAACCAGGTGGAAGCGT	
QPtoC3H3-F	TCTACGCTTCAAGCTCCCACCA	
QPtoC3H3-R	GGAACCTCACAGGCTTGACG	
QPtoCCoAOMT1-F	CAGTAATTCAGAAAGCTGGTGTTGC	
QPtoCCoAOMT1-R	GCATCCACAAAGATGAAATCAAAAC	
QPtoCCR2-F	CGGTGATTCAGAAAGCTGGTCTGGA	
QPtoCCR2-R	GCATCCACAAAGATGAAGTCATAAG	
QPtoF5H2-F	TCTGCGTCATGAAGCTATTTAGCC	
QPtoF5H2-R	TGTCTTAAGCATTGAGTCCACCTCA	
QPtoCOMT2-F	TCTTGAAGAATTGCTATGACGCCT	
QPtoCOMT2-R	GAATGCACTCAACAAGTATCACCTTG	
QPtoCAD1-F	AAGTTTGTGGTGAGAATTCCTGATG	
QPtoCAD1-R	AAACTGTCAATCCAGCGCACAATAG	
PtoLAC40-F	AAGGCGGTTTCACTTTGCCAGTCC	
PtoLAC40-R	TAGGTGGCATCAACTTCCACGACA	
PtoPO6-F	CTACTGTTATTGCTGGAGGTCC	
PtoPO6-R	TGTGGCTCCCAGATAGTGCTAC	
PtoGT43B-F	TTGAGGTGTTTGGAACATGGC	
PtoGT43B-R	AGTTCTGTGAGGTTTGCTGGAC	
PtoCesA17-F	CCCCTCTAGTCACGGGCAACACAC	
PtoCesA17-R	AAGGTGCACATTGAAGCACCATCG	
PtoCesA18-F	GTTGGCCTCTGTCTTCTCTCTTGTT	
PtoCesA18-R	CAATCAATGGAAATGCAGGTCTCCG	
PtrCesA2B-F	AGGTTAAGATGGAGCGG	
PtrCesA2B-R	ACGAGGTTGATGATCAAGCC	
SubPtoMYB156-F	ACACCGCACACCCTTCTAAG	Primers for Subcellular Localization
SubPtoMYB156-R	TCCCTTTCTCCTTTCTCTCC	
ProPtrGT43B-F	ATATTCTCAACACTATAGTCATTG	Primers for GUS activity assay (Promoters)
ProPtrGT43B-R	GCTAAACCCCTCAAAAACGTG	
pro-ptrC4H2-F2	GGGGTACCAGATGGGCATGCAGGAGTTG	
pro-ptrC4H2-R2	ACTGCAGCCAAAGGAGTACTGAAAGAG	
pro-ptrC3H3-F2	GGGGTACCACTTGGTGGATTTGAGACCAC	
pro-ptrC3H3-R2	ACTGCAGTCTGAAGGGCCTTGGCAGAAT	
ProPtrCesA-F	GGAGAGGCTACACTGTCCAGATGGAG	
ProPtrCesA-R	CTGGCTTCCATGTTGAGCAATGG	

SupplementaryTable S2. Anatomical properties of wild-type and transgenic *35S:PtoMYB156* poplar.

Line	Area of Xv	Area of Xf (μm^2)	Area of X (μm^2)	Ratio of X area/STS	Area of P (μm^2)	Ratio of P area/STS
WT	1248.92±89.02	298.72±22.70	61412.10±7014.53	31.94±2.23%	3778.50±12.22	5.17±0.76%
L4	1200.29±55.21	254.20±21.10	52276.30±118.30	27.34±5.37%	2128.32±17.36*	3.99±0.86%

Xv, xylem vessel cell; Xf, xylem fiber; X, xylem; STS, stem transverse section area; P, phloem.

SupplementaryTable S3. Measurements of secondary cell wall thickness of wild type and transgenic *35S:PtoMYB156* poplar.

Sample	Xylem vessel (μm)	Xylem fiber (μm)
WT	3.78±0.618	4.18±0.50
L4	3.53±0.89*	2.93±0.69**

Student's *t* test: *, $P < 0.05$; **, $P < 0.01$.

SupplementaryTable S4. Syringyl, guaiacyl and p-hydroxyphenyl monomer composition, and total monomer yield of wild-type and transgenic *35S:PtoMYB156* plants as determined by HPLC.

Sample	Klason lignin (mg/g)	H (%)	G (%)	S (%)	S/G
WT	245±8.7	0.24±0.02	42.32±2.33	57.44±2.71	1.36±0.01
L4	211.9±10.1*	0.85±0.09	42.34±2.88	56.81±4.20	1.35±0.01

Student's *t* test: *, $P < 0.05$.
